# The Best School Discipline

**Published:** 1920-11-13

**Authors:** 


### The Best School Discipline.

An address given by Mr. Rowe, headmaster of the Manchester Street L.C.C. School, on "The Value of Recreation Outside the School," and quoted at length in the "Charity Organisation Review," is in accordance with the views frequently expressed in these columns. "In the schoolroom," Mr. Rowe points out, "there is very little outlet for the physical energies of the average child. He is often required to subdue and control his natural 'active' impulses, and become more or less 'passive' and receptive, or at least quiescent, over fairly long periods of time. Sometimes the steam acquires so good a head as to be near the danger point. Most teachers and parents know of daring escapades arising out of these pent-up forces, and it is well for the child if the true cause is recognised, and not put down to innate perversity. Some natures must of sheer necessity react strongly from the tedium of sustained humdrumness of much that goes by the name of teaching. Luckily for these children, and also for the average child, it is being increasingly held that recreative games are the natural corrective and balance to the dolours of the schoolroom, especially those games and contests that require bodily activity.

"Failing these, the child will play spontaneously, and gain physical relief by indulging in unorganised romping, pushing, shouting, dancing, and squealing. This is the time when the myriads of body cells—not under control of the child's will—obtain their finest opportunity to function—*i.e.*, to grow and to divide, an activity upon which all animal development depends. With boys there is no difficulty in getting them to play games, and to play them earnestly and well. In fact, where a good healthy sportsmanlike spirit is developed by tactful supervision, the moral and social nature of a child may be stimulated in no other equally valuable way. The commandment 'to play the game' may readily become the boy's decalogue. Class-room studies and even the more active manual occupations are largely individualistic in their tendencies; but in games and contests not only are courage, patience, and hardihood demanded, the power to take a beating with a smile, and to win without a display of triumph or conceit, but each member of a team must work in friendly rivalry with the others, under a properly constituted authority, for the common weal. The young player will willingly submit to a discipline that can be obtained in no other way in order that his side may win, and under the eyes of his peers the evidence of hard knocks is sternly repressed.

Games are essentially competitive, but they are essentially co-operative too, and this constitutes their high value as moral and social training. For in the strife of opposing skill and intelligence valuable knowledge of human nature is acquired, and also tact and judgment in dealing with one's fellows.

"More might be done, I think, in the schoolroom to render the fine corporeal habits of the playing-field more real and intelligent to the players, and to show their connection with real life and events."

